# Road Use Pattern and Street Crossing Habits of Schoolchildren in India

**DOI:** 10.3389/fpubh.2021.628147

**Published:** 2021-02-05

**Authors:** Rajnarayan R. Tiwari, Shruti Patel, Annie Soju, Prarthana Trivedi

**Affiliations:** ^1^National Institute for Research in Environmental Health, Bhopal, India; ^2^ENVIS, National Institute of Occupational Health, Ahmedabad, India

**Keywords:** road traffic injuries, school children, street crossing, road use, India

## Abstract

Road traffic accidents (RTAs) contribute significant DALYs in the global burden of diseases. Vulnerable groups particularly pedestrians and children are at an increased risk. Road use pattern, street crossing habits, and road safety awareness are important determinants of RTAs. The present study was carried out to assess the road use pattern and street crossing habits of schoolchildren. This cross-sectional study included 497 schoolchildren of 12–15 years. The interview technique was used as a tool for data collection on a predesigned questionnaire. A total of 40.4% of schoolchildren did not like to go to school alone and wanted somebody from the family to drop them to school. About one quarter of the students were afraid of traffic and expressed their inability to deal with traffic on the road. A total of 10.7% reported crossing the street in groups, and 1.4% reported running while crossing the street. Only 80.9% of students received some form of road safety training, and the parents and schools were the major source of information for such safety training. Age <14 years and a lower level of mother's education were found to be significant contributors for poor road crossing habit in univariate as well as multivariate analysis. The study suggests that the knowledge regarding safe road use and street crossing was lacking among study participants albeit in a small proportion only. Safety aspects can be partly strengthened by imparting practical knowledge about road use pattern, street crossing habits, and road safety procedures.

## Introduction

Increasing economic development has resulted in the overall development of the road network as well as vehicle technology. In countries like India, this has meant increased vehicle density on roads thereby increasing possibilities for road traffic accidents (RTAs). A recently published burden-of-disease study reported that RTAs are in the top 15 accounting for the most Disability-Adjusted Life Years (DALYs). It contributed 3.3% of DALYs at all ages and from Years of Life Lost (YLLs), i.e., due to mortality (1). India had an increase in the number of deaths due to road injuries of 58.7% between 1990 and 2017. Similarly, the proportion of deaths due to road injuries among all deaths in India increased from 1.7% in 1990 to 2.2% in 2017 (2). Thus, India had 17.6% of the total global deaths due to road injuries in 2017. Road injuries were the leading cause of death in males aged 15–39 years age in 2017. In Gujarat, the crude death rate was 13.5 per lakh population which was an increase of 3.1% from 1990 to 2017. This suggests the gravity of RTAs.

Further, non-fatal RTAs have other consequences such as physical, psychological, everyday life, and financial consequences. The physical consequences of RTAs can be in the form of temporary or permanent disability that will not only hamper daily activities. In a study, it was found that even minor and moderate injuries had detrimental long-term health consequences. The psychological consequences include travel anxiety and posttraumatic stress disorders, which sometimes force life alteration (3). For children, it is reported that psychological consequence was associated with disability, especially for travel (4). The costs of fatality and injuries due to RTAs have a tremendous impact on social well-being and socioeconomic development endeavors (5).

Vulnerable road users such as pedestrians, cyclists, and children are common victims of RTAs. Pedestrian deaths accounted for 35.1% of all deaths due to road injuries in India in 2017 (2). One of the important factors for the occurrence of RTA is the road use pattern and behavior of children. Schoolchildren can either be escorted to school by parents or elders or may be allowed to go alone to school. Escorting them to school means a lot of household travel as the peak traffic density coincides with the beginning and end of the school day (6). If they are allowed to go alone, the high level of risk behaviors such as waiting in the street as opposed to on the sidewalk before crossing, non-compliance with traffic lights, crossing outside the crosswalk, or distraction during the different stages of street crossing is worrisome (7). These risky behaviors can be curbed through educational measures to teach proper road use patterns and the right way to cross the street habits particularly in this vulnerable group. The present study was carried out in Ahmedabad which is a mega city in the western state of Gujarat in India. It has an area of 466 square kilometers with a population of 5.5 million. It has grown in the form of circular rings around the walled city area with a total asphalted road network length of 2,399 km. Only 40% of the total road network has sidewalks, while for the remaining no sidewalks are present. Though the maximum speed limit within the city is 40 kmph, the peak hour speed is 25 kmph. The city had 4.81 million vehicles in 2019 with an annual increment rate of 5–6%. In the year 2019, 1,371 RTAs were registered by city traffic police that claimed the lives of 423 individuals (8). With this background, the present study is carried out among schoolchildren to understand their road use behavior, practice during street crossing, and knowledge regarding safety precautions to be taken while on the street.

## Materials and Methods

The present cross-sectional study was carried out among schoolchildren in the age group of 12–15 years visiting the institute as part of their educational visit to the ENVIS Centre of National Institute of Occupational Health, Ahmedabad. Fifteen different schools from the nearby regions visited during the study period. These included six government-run schools and nine schools run by private trusts and partly aided by government. The schoolchildren visited the ENVIS Centre as per their educational program, and thus the participating children were random participants of the study. A total of 497, schoolchildren were enrolled in the study which comprised 254 boys and 243 girls. After gaining ethical clearance from the Institutional Ethics Committee and informed verbal assent from the students, the information was recorded on the predesigned questionnaire.

The questionnaire was developed after reviewing similar studies available in the literature. It consisted of three major sections. The first section was about the demographic information of the participants. The second section included the road use pattern of the participants such as perception of school distance from home, mode of reaching school, perception about the traffic and way of dealing with it, and knowledge about the health effects of noise. The third section included questions regarding street crossing habits and safety education such as perception of difficulty in crossing the road, place of crossing the road, risky way of crossing roads such as running while crossing or crossing in groups, and sources of receiving safety education, if received.

Responses to the questions were dichotomized and graded on a scale of “0” and “1” with “1” indicating a correct response and “0” being an incorrect response. The score for all the responses for each individual was summed up for road use pattern and street crossing habits taken together. The mean score was compared according to study variables age, sex, mother's education, and father's education. For comparing the mean score, the study variables were dichotomized into age <14 (*n* = 379) and ≥ 14 (*n* = 118) years, government (*n* = 104) or private (*n* = 389) schools, father's education into primary level or below (*n* = 114) and middle school or above (*n* = 383), and mother's education into primary level or below (*n* = 231) and middle school and above (*n* = 266). There were eight questions regarding street crossing habits, and thus, a maximum of eight marks could be scored. Thus, assuming an arbitrary cutoff score of five, the scores obtained were categorized into low (scoring <5) and adequate (scoring ≥5). The statistical analysis was carried out using SPSS 15.0 and included the calculation of percentages and proportions and the application of test of significance such as chi-square. For multivariate analysis, age ≥14 years, male gender, higher parent's education, and private school were considered as reference categories.

## Results

The present study included 497 students from primary and middle schools. A total of 51.1% of students were boys while only 48.9% were girls. The mean age of boys was 13.5 ± 1.2 years while that for girls was 13.7 ± 1.1 years. This difference was statistically non-significant (*t* = 3.28; df = 1; *p* = 0.07). Using the modified Kuppuswamy scale (9), which takes into account the occupation and education of the head of the family and per capita income, it was found that the majority of the students belonged to lower middle socio-economic strata.

The age-wise and gender-wise distribution of road use perception and pattern of the schoolchildren is shown in Table 1. A total of 220 (44.3%) students felt that their school was far from their home. It was found that the perception that school was at a distance did not vary significantly according to age groups (χ^2^ = 1.5, df = 1, *p* = 0.24) and gender (χ^2^ = 0.9, df = 1, *p* = 0.37). When asked about the way they reach their school, 59.6% mentioned that they would walk on their own. However, there were about 19.5% of students who were accompanied by their parents or neighbors. When the difference in proportion of those reaching school on their own was compared with those reaching school accompanied by others, it was found that the difference was statistically non-significant according to age (χ^2^ = 0.13, df = 1, *p* = 0.71) but a significantly larger number of girls were accompanied by others when compared to boys (χ^2^ = 7.2, df = 1, *p* = 0.007). Of those reaching school alone, a significantly greater number of boys were reaching school by cycling or on two-wheelers as compared to girls (χ^2^ = 10.6, df = 1, *p* = 0.014). A total of 201 (40.4%) did not like to go to school alone and wanted somebody from the family to drop them to school. This included a greater number of those aged <14 years (χ^2^ = 68.5, df = 1, *p* = 0.00) and girls (χ^2^ = 7.2, df = 1, *p* = 0.007). About one quarter of the students were afraid of traffic and expressed their inability to deal with traffic on the road, and this included mostly those aged <14 years. Although 88.5% felt that vehicular noise is harmful to health, only 58.9% had correct knowledge of harm caused by noise. The correct responses included distraction, irritation, deafness, and effect on mental health. A significantly greater number of girls had correct knowledge about the harms of vehicular noise as compared to boys (χ^2^ = 6.8, df = 1, *p* = 0.01).

**Table 1:** Age group-wise and gender-wise distribution of road use perception and pattern of schoolchildren.

**Characteristic**	**Age group (in years) (*****N*** **=** **497)**			**Gender (*****N*** **=** **497)**		
	**<14** ***n* = 379**	**≥14*n* = 118**	***p*-Value**	**Males*n* = 254**	**Females** ***n* = 243**	***p*-Value**
**Feels that school is at distance**						
Yes No	162 (42.7) 217 (57.3)	58 (49.2) 60 (50.8)	χ^2^= 1.5 *p* = 0.24	107 (42.1) 147 (57.9)	113 (46.5) 130 (53.5)	χ^2^= 0.9 *p* = 0.37
**Means of reaching school**						
On my own By bus/van With parent in car With parent on foot With others on foot	224 (59.1) 77 (20.3) 29 (7.6) 9 (2.4) 40 (10.6)	72 (61.0) 27 (22.9) 8 (6.8) 3 (92.5) 8 (6.8)	χ^2^= 0.13 *p* = 0.71	166 (65.4) 54 (21.3) 9 (3.5) 7 (2.8) 18 (7.1)	130 (53.5) 50 (20.6) 28 (11.5) 5 (2.1) 30 (12.3)	χ^2^= 7.24 *p* = 0.007
**If own, means**^**#**^						
On foot By bicycle By two wheeler	191 (85.2) 28 (12.5) 5 (2.2)	66 (91.7) 6 (8.3) -	χ^2^= 2.9 *p* = 0.41	139 (54.7) 23 (9.1) 4 (1.6)	118 (48.6) 11 (4.5) 1 (0.4)	χ^2^= 10.6 *p* = 0.014^*^
**Like to go to school on own**						
Yes No	224 (59.1) 155 (40.9)	72 (61.0) 46 (39.0)	χ^2^= 68.5 *p* = 0.00^*^	166 (65.4) 88 (34.6)	130 (53.5) 113 (46.5)	χ^2^= 7.2 *p* = 0.007^*^
**Afraid of traffic**						
Yes No	107 (28.2) 272 (71.8)	22 (18.4) 96 (81.6)	χ^2^= 4.3 *p* = 0.04^*^	60 (23.6) 194 (76.4)	69 (28.4) 174 (71.6)	χ^2^= 1.5 *p* = 0.26
**Deal with traffic movement on own**						
Yes No	299 (78.9) 80 (21.1)	108 (91.5) 10 (8.5)	χ^2^= 9.7 *p* = 0.002^*^	208 (81.9) 46 (18.1)	199 (81.9) 44 (18.1)	χ^2^= 0.9 *p* = 0.55
**Feel vehicular noise is harmful**						
Yes No	332 (87.6) 47 (12.4)	108 (91.5) 10 (8.5)	χ^2^= 1.4 *p* = 0.32	223 (87.8) 31 (12.2)	217 (89.3) 26 (10.7)	χ^2^= 0.6 *p* = 0.67
**Which harm is caused by**						
**vehicular noise**						
Correct response Incorrect response	160 (42.2) 219 (57.8)	43 (36.4) 75 (63.6)	χ^2^= 1.2 *p* = 0.29	136 (53.5) 118 (46.5)	158 (65.0) 85 (35.0)	χ^2^= 6.8 *p* = 0.01^*^

* *Statistically significant*.

# *includes only those reaching school on their own*.

The street crossing habits and its distribution according to age groups and gender are depicted in Table 2. Only 112 (22.5%) responded that crossing the street is a difficult task. When this perception was further analyzed, it was found that the difference was statistically non-significant according to age group (χ^2^ = 0.2, df = 1, *p* = 0.8) as well as gender (χ^2^ = 0.7, df = 1, *p* = 0.8). Although the majority (86.5%) looked for crossing places like zebra crossing or crosswalks for crossing the road, 67 (13.5%) schoolchildren preferred crossing from any spot on the road. This included a significantly greater number of those aged <14 years (χ^2^ = 9.4, df = 1, *p* = 0.002) and boys (χ^2^ = 13.1, df = 1, *p* = 0.00). A total of 227 (45.7%) schoolchildren used to hold other persons' hands during street crossing while 27 (5.4%) never crossed the road on their own. Although the majority (87.9%) of the students had correct street crossing habits such as looking while crossing, making sure that traffic is far enough away, and continuing to look while crossing, still 53 (10.7%) reported crossing the street in groups and 7 (1.4%) reported running while crossing the street. When the activities during street crossing were dichotomized into correct and incorrect and compared according to age groups and gender, the difference was found to be statistically non-significant for both the variables.

**Table 2:** Age group-wise and gender-wise distribution of street crossing habits of schoolchildren.

**Characteristic**	**Age group (in years) (*****N*** **=** **497)**			**Gender (*****N*** **=** **497)**		
	** <14** ***n* = 379**	**≥14*n* = 118**	***p*-Value**	**Males*n* = 254**	**Females** ***n* = 243**	***p*-Value**
**Crossing the street is**						
Easy Difficult	292 (77.0) 87 (23.0)	93 (78.8) 25 (21.2)	χ^2^= 0.2 *p* = 0.8	198 (78.0) 56 (22.0)	187 (77.0) 56 (23.0)	χ^2^= 0.7 *p* = 0.8
**Look for crossing place before crossing**						
Yes No	318 (83.9) 61 (16.1)	112 (94.9) 6 (5.1)	χ^2^= 9.4 *p* = 0.002^*^	206 (81.1) 48 (18.9)	224 (92.2) 19 (7.8)	χ^2 =^ 13.1 *p* = 0.00^*^
**Hold another person's hand while crossing**						
Yes No	167 (44.1) 212 (55.9)	60 (50.8) 58 (49.2)	χ^2^= 1.7 *p* = 0.21	111 (43.7) 143 (56.3)	116 (47.7) 127 (52.3)	χ^2^= 0.8 *p* = 0.37
**Frequency of crossing the street on own**						
Always Sometimes Never	162 (42.7) 196 (51.7) 21 (5.6)	58 (49.2) 54 (45.7) 6 (5.1)	χ^2^= 1.5 *p* = 0.5	125 (49.2) 117 (46.1) 12 (4.7)	95 (39.1) 133 (54.7) 15 (6.2)	χ^2^= 5.2 *p* = 0.07
**Do while crossing the street**						
Look to cross Make sure traffic is far enough away Continue to look while crossing Cross in a group with other children Run	92 (24.3) 108 (28.5) 132 (34.8) 43 (11.3) 4 (1.1)	63 (53.4) 11 (9.3) 31 (26.3) 10 (8.5) 3 (2.5)	χ^2 =^ 108.9 *p* = 0.00^*^	74 (29.2) 57 (22.4) 90 (35.4) 28 (11.0) 5 (2.0)	81 (33.4) 62 (25.5) 73 (30.0) 25 (10.3) 2 (0.8)	χ^2^= 24.4 *p* = 0.04^*^
**Received traffic safety education**						
Yes No	314 (82.8) 65 (17.2)	107 (90.7) 11 (9.3)	χ^2^= 4.3 *p* = 0.04^*^	200 (78.7) 54 (21.3)	221 (90.9) 22 (9.1)	χ^2^= 14.3 *p* = 0.00^*^
**Source of traffic safety education**^**$**^^**#**^						
Parents School TV Internet Others	102 (32.5) 180 (57.3) 36 (11.5) 11 (3.5) 21 (6.7)	65 (60.7) 74 (69.2) 20 (18.7) 6 (5.6) 11 (10.3)	χ^2^= 4.5 *p* = 0.33	75 (29.5) 108 (40.9) 32 (12.6) 8 (3.1) 11 (4.3)	92 (37.9) 146 (60.1) 24 (9.9) 9 (3.7) 21 (8.6)	χ^2^= 5.4 *p* = 0.24

* *Statistically significant*.

$ *Included only those who received safety education*.

# *Multiple responses*.

Only 421 (80.9%) students responded that they have received road safety training. A significantly greater number of those aged ≥14 years (χ^2^ = 4.3, df = 1, *p* = 0.04) and girls (χ^2^ = 14.3, df = 1, *p* = 0.00) responded to having received safety education. When these students who have received safety education were asked about the source of such training, most mentioned their parents and their schools as the source of such training.

The mean scores of street crossing habits according to study variables are depicted in Table 3. After assessing the response of each participant as correct or incorrect, scores were given on a scale of “1” or “0,” respectively. The mean scores were compared with the dichotomized study variables, namely, age, type of school, father's education, mother's education, and gender. The lesser the mean score, the more inappropriate the street crossing habit. It can be observed that those aged <14 years (*t* = 7.85; *p* = 0.005), studying in government schools (*t* = 22.17; *p* = 0.000), with a father educated up to primary school or less (*t* = 6.75; *p* = 0.01), and with a mother educated up to primary school or less had significantly lower scores as compared to their counterparts.

**Table 3:** Mean scores of street crossing habit according to study variables.

**Study variable**	**N**	**Mean score ±SD**	***t*; *p*-Value**
**Age (in years)**			
<14≥14	379 118	6.32 ± 1.346.70 ± 1.06	7.85; 0.005^*^
**Gender**			
MaleFemale	254 243	6.35 ± 1.316.49 ± 1.27	1.44; 0.23
**Mother's education**			
Primary and belowMiddle and above	231 266	6.19 ± 1.386.61 ± 1.17	12.79; 0.000^*^
**Father's education**			
Primary and belowMiddle and above	114 383	6.14 ± 1.496.50 ± 1.22	6.75; 0.01^*^
**School type**			
GovernmentPrivate	104 389	5.89 ± 1.476.55 ± 1.21	22.17; 0.000^*^

* *Statistically significant*.

The scores of street crossing habits were further dichotomized as low score (<5) or high score (≥5). This dichotomized variable was used for the multivariate analysis with study variables (Table 4). It was observed that those aged <14 years had 2 times higher odds of getting lower scores (OR = 2.0; 95% CI = 1.14–3.52). Similarly, children whose mother had a lower level of education had a 1.8 times higher chance of getting a low score (OR = 1.8; 95% CI = 1.12–2.90), indicating poor knowledge about the correct street crossing practices.

**Table 4:** Multivariate analysis between street crossing scores and study variables.

**Study variable**	**Crude OR (95% CI)**	***p*-Value**	**Adjusted OR (95% CI)**	***p*-Value**
**Age (in years)**				
≥14 <14	Reference 1.88 (1.09–3.23)	0.02	Reference 2.00 (1.14–3.52)	0.016^*^
**Gender**				
MaleFemale	Reference 1.13 (0.75–1.71)	0.57	Reference 1.03 (0.67–1.64)	0.88
**Mother's education**				
Middle and above Primary and below	Reference 1.66 (1.09–2.52)	0.017	Reference 1.8 (1.12–2.90)	0.016^*^
**Father's education**				
Middle and above Primary and below	Reference 1.28 (0.79–2.06)	0.31	Reference 0.85 (0.49–1.48)	0.56
**School type**				
PrivateGovernment	Reference 0.63 (0.39–1.02)	0.059	Reference 0.73 (0.43–1.22)	0.23

* *Statistically significant*.

## Discussion

The present study was carried out to assess the street crossing habit and road use pattern of schoolchildren. Both aspects are important as pedestrians accounted for 13% of those killed in accidents in 2017 in India, clearly emphasizing the role of safe road use patterns (10). The majority of the students in the study were walking to school and were not accompanied by any elders. Only 6.8% of children were going by bicycle, and the number of girls using a bicycle was lower than the boys. This may be because in Indian society boys are given more freedom and are less restrained by the parents to move around as compared with girls (11–13). Also, using a bicycle in young boys may result in a higher risk of road traffic injuries as a result of collision with another vehicle and due to skidding/falling from the bicycle (14).

When asked about street crossing habits, approximately 1 in 4 students found crossing the street difficult due to heavy vehicle rush. In total 83.9% responded that they looked for a place to cross such as zebra crossings or crosswalks, suggesting that nearly one-sixth did not look for any such crossing spot and thus had bad habits of crossing the road from any spot on the road. Similarly, 1 in 6 students were crossing the street in groups or by running which are considered to be bad road etiquette. Earlier studies also reported that the majority of pedestrians were not using crosswalks for crossing the streets (7, 15). However, another study shows that a zebra crossing was chosen as the most favorable type of crossing facility by the majority of pedestrians (16).

Despite that road etiquette and safety education are important for controlling RTAs especially involving pedestrians, it was important to note that 80.9% received some form of traffic safety education and nearly one-fifth did not receive any traffic safety education. The major sources for such education were parents and school. While parents might have taught road etiquette practically while accompanying the child on the street, the knowledge gained through schoolbooks might be theoretical instead of practical. It has been widely accepted that better road safety knowledge and the avoidance of walking- or cycling-related risk behaviors are protective factors for road traffic injuries especially involving children (17). Also, many injuries to children cannot be prevented without some degree of active behavior on the part of parents. Thus, more injury prevention programs are needed to improve road safety knowledge and reduce risk behaviors.

Further, an inadequate street crossing habit was found to be associated with younger age and a lower level of mother's education. Maternal grade attainment and literacy are associated with a wide range of preventive and treatment-oriented health behavior and the effective use of health services (18). Similarly, lower-level mother's education can result in the poor street crossing attitudes of schoolchildren. In addition, it is not uncommon for children to play on roads in India as not many neighborhoods have playgrounds. Also, these residential areas often do not have speed or traffic volume restrictions, thereby increasing the risk of road traffic injuries for children. They are often unsupervised while playing or running errands, and parental supervision can reduce the RTI risk in children (14). The role of age can be explained by the fact that due to inexperience and immaturity, younger children are unable to take decisions on street crossing.

This study had several limitations. The RTAs are due to multiple factors, of which road safety education is one important factor. Information about other factors would have given a comprehensive perspective. However, due to a feasibility issue it could not be done. Secondly, most of the information about safety education was self-reported and thus is associated with inherent bias such as confirmation bias. Thirdly, after categorization of the variables, the effective sample size for some of the categories was small and thus the generalization of results should be done with precaution.

Thus, the study suggests that knowledge regarding safe road use and street crossing was lacking among study participants albeit only in a small proportion. Safety aspects can be partly strengthened by imparting practical knowledge about road use patterns, street crossing habits, and road safety procedures. In India, several cities such as Nagpur, Chandigarh, and Hyderabad have children's traffic parks in which children learning the rules of the road while driving toy vehicles can be an effective source to impart additional practical awareness along with the theoretical knowledge gained through textbooks and parents as reported in the present study. The park has roads with traffic lights, pictorial representations of safety slogans, signals, rules, and dummy traffic police to teach children safe traffic behavior which can inculcate good traffic habits from a very early age. Also, while advocating active commuting to school which may increase children's daily physical activity and help them maintain a healthy weight, a multipronged strategy including communities that support independent mobility by providing child-friendly social and built environments, safety from traffic, and policies that promote local schools and safe vehicle-free zones around school should be adopted (19–21). Although literature on safe vehicle-free zones around school is missing, several media reports suggest that in Glasgow, UK, a pilot project has been initiated in August 2019. The effectiveness and possibilities of such a solution need to be cautiously examined. Similarly, a School Safety Zone project has been initiated in one of the schools of Bengaluru, Southern India, where traffic and the speed of vehicles in the school environment are regulated using signage systems by the schoolchildren. In another literature review on the street, closure schemes have shown that there is an improvement in perception of road safety by such schemes (22).

## Data Availability Statement

The raw data supporting the conclusions of this article will be made available by the authors, without undue reservation.

## Ethics Statement

The studies involving human participants were reviewed and approved by IEC of ICMR-National Institute of Occupational Health Ahmedabad. Written informed consent to participate in this study was provided by the participants' legal guardian/next of kin.

## Author Contributions

RT: data analysis, manuscript preparation, and review. SP: data collection, data entry, and manuscript review. AS: data collection, data entry, and manuscript review. PT: data collection, data entry, and manuscript review. All authors contributed to the article and approved the submitted version.

## Conflict of Interest

The authors declare that the research was conducted in the absence of any commercial or financial relationships that could be construed as a potential conflict of interest.
